# Carbonic Anhydrase IX Controls Vulnerability to Ferroptosis in Gefitinib-Resistant Lung Cancer

**DOI:** 10.1155/2023/1367938

**Published:** 2023-01-31

**Authors:** Chen Zhang, Xiyi Lu, Xinyin Liu, Jiali Xu, Jun Li, Tianyu Qu, Jiali Dai, Renhua Guo

**Affiliations:** Jiangsu Province Hospital and Nanjing Medical University First Affiliated Hospital, Nanjing, Jiangsu, China

## Abstract

Acquired resistance to epidermal growth factor receptor tyrosine kinase inhibitors (EGFR-TKI, such as gefitinib) in lung cancer continues to be a major problem. Recent studies have shown the promise of ferroptosis-inducing therapy in EGFR-TKI resistant cancer, but have not been translated into clinical benefits. Here, we identified carbonic anhydrase IX (CA9) was upregulated in gefitinib-resistant lung cancer. Then we measured the cell viability, intracellular reactive oxygen species (ROS) levels, and labile iron levels after the treatment of ferroptosis inducer erastin. We found that CA9 confers resistance to ferroptosis-inducing drugs. Mechanistically, CA9 is involved in the inhibition of transferrin endocytosis and the stabilization of ferritin, leading to resistance to ferroptosis. Targeting CA9 promotes iron uptake and release, thus triggering gefitinib-resistant cell ferroptosis. Notably, CA9 inhibitor enhances the ferroptosis-inducing effect of cisplatin on gefitinib-resistant cells, thus eliminating resistant cells in heterogeneous tumor tissues. Taken together, CA9-targeting therapy is a promising approach to improve the therapeutic effect of gefitinib-resistant lung cancer by inducing ferroptosis.

## 1. Introduction

Non-small cell lung cancer (NSCLC) is one of the most common cancers worldwide with high mortality rate [1]. With the advent of molecular targeted therapies, survival in NSCLC continues to improve. Epidermal growth factor receptor (EGFR) is a critical molecular target in NSCLC patients. Although EGFR-tyrosine kinase inhibitors (EGFR-TKI, such as gefitinib or erlotinib) have resulted in significant clinical benefit in patients with EGFR-mutant NSCLC [2, 3], acquired resistance inevitably develops [4]. Multiple mechanisms of gefitinib resistance have been reported, including the EGFR T790M mutation, MET amplification, ERBB2 amplification, and cancer phenotypic transformation [5–7]. Therapeutic options could be developed according to the resistance mechanisms, such as adopting osimertinib or combining with MET inhibitors. However, the resistance mechanisms of gefitinib have yet to be discovered in approximately 20% of NSCLC patients [8]. Therapeutic options are limited in these patients.

Ferroptosis is a nonapoptotic form of cell death that is iron-dependent. Ferroptosis was first found to be triggered by erastin, a RAS inhibitor, which is characterized by intracellular redox imbalance and increased levels of reactive oxygen species (ROS) [9]. Recently, the therapy-resistant cell state in cancer cells has been reported to be vulnerable to ferroptosis [10]. You et al. reported that erlotinib-tolerant persister head and neck cancer cells are vulnerable to ferroptosis by GPX4 or xCT inhibition [11]. Zhang et al. found that the histone deacetylase inhibitor vorinostat combined with erastin could suppress the viability of EGFR-TKI-resistant lung cancer cells by inducing ferroptosis [12]. These results show the promise of ferroptosis-inducing therapy in EGFR-TKI resistant cancer cells.

However, there are outstanding questions that remain to be addressed before the practical application of ferroptosis-inducing therapy. For example, most of the ferroptosis-inducing agents were only examined in cultured cell lines, which have not been translated into clinical benefits [13]. As a common inducer of ferroptosis, cisplatin is the most frequently used agent for lung cancer in clinical practice [14]. Cisplatin-based chemotherapy shows certain efficacy for NSCLC patients after EGFR-TKI resistance. In addition, cisplatin-based chemotherapy has been reported to eliminate EGFR-TKI resistant cells, thus creating beneficial conditions for the retreatment of EGFR-TKI [15]. We speculated that cisplatin could play a role in eliminating drug-resistant cells by inducing ferroptosis. However, the long-term benefits of EGFR-TKI retreatment after cisplatin-based chemotherapy are still limited [15]. It is important to identify what genetic alterations in EGFR-TKI resistant cells may contribute to the vulnerability to ferroptosis. Thus, we can find ways to induce ferroptosis in resistant cancers more effectively.

Carbonic hydrase IX (CA9), a ferroptosis-related gene, was found to be upregulated in gefitinib-resistant cells in our study. CA9 is a member of the carbonic anhydrase family, which catalyzes the reversible hydration of carbon dioxide to maintain intracellular pH homeostasis [16]. A recent study showed that the upregulation of CA9 significantly inhibits tumor cell ferroptosis under hypoxia [17]. The abnormal expression of CA9 has been reported to affect the treatment efficacy in NSCLC [18–20]. In addition, elevated CA9 expression is closely related to poor prognosis in EGFR-mutant lung cancer [21]. Although CA9 is highly expressed in EGFR-TKI resistant NSCLC cells, as a marker of hypoxia [22, 23]. The role of CA9 in regulating ferroptosis remains unknown.

In the present study, we suggest that CA9 confers resistance to ferroptosis in gefitinib-resistant lung cancer cells by regulating iron metabolism. Inhibiting CA9 is a promising approach to improve lung cancer treatment by targeting ferroptosis.

## 2. Materials and Methods

### 2.1. Cell Culture

The human non-small-cell lung cancer cell lines PC9 (EGFR exon 19 deletion) and HCC827 (EGFR exon 19 deletion), and the normal human bronchial epithelial cell lines (Beas2B and HBE) were purchased from Shanghai Institute of Biochemistry and Cell Biology, Chinese Academy of Sciences (Shanghai, China). The gefitinib-resistant cell lines PC9/GR and HCC827/GR were established by exposing PC9 and HCC827 cells to increasing concentrations of gefitinib (HY-50895, MCE, China) as previously described [24]. The parental cells were cultured in the medium containing 0.3 *μ*M gefitinib until they could survive in the medium containing 1.0 *μ*M gefitinib. During this process, the drug-containing medium was replaced twice per week. Then the resistant cells were maintained in 1.0 *μ*M gefitinib. All cells were cultured in RPMI-1640 medium supplemented with 10% fetal bovine serum.

### 2.2. Clinical Samples Collection

Clinical tissues and peripheral blood were acquired from the First Affiliated Hospital of Nanjing Medical University. Collection of samples and clinical information was undertaken with ethical review board approval (No. 2019-SRFA-226).

### 2.3. Cell Counting Kit-8 (CCK8) Assay

The CCK8 assay was performed according to the manufacturer's instructions for the CCK8 kit (HY-K0301, MCE, China). Briefly, cells were plated in 96-well plates and treated with various concentrations of gefitinib, erastin (HY-15763, MCE, China), U104 (HY-13513, MCE, China), z-VAD (HY-16658B, MCE, China), ferrostatin-1 (Fer-1, HY-100579, MCE, China) and desferrioxamine mesylate (DFO, HY-B0988, MCE, China) for 48 h. Ten microliters of CCK8 reagent was added to each well and incubated for 1 h at 37°C. Then, the plates were measured by a microplate reader at 450 nm.

### 2.4. Colony Formation Assay

Cells were plated in 6-well plates at a density of 3000 cells/well and incubated overnight. Then, the medium was replaced twice a week for approximately 10 days. The colonies were stained with a 0.1% crystal violet solution (C0121, Beyotime, China). Visible colonies that were larger than 0.5 mm were counted.

### 2.5. Measurement of ROS

The level of intracellular ROS was measured according to the manufacturer's instructions for the ROS assay kit (S0033S, Beyotime, China). Briefly, cells were plated into 6-well plates and stained with DCFH-DA (10 *μ*M) for 20 min at 37°C. Then, the cells were harvested, and the fluorescence was detected by flow cytometry (FITC channel) to measure the intracellular ROS.

### 2.6. Measurement of Labile Iron

The level of intracellular labile iron was measured according to the manufacturer's instructions of the FerroFarRed kit (GC903-01, Goryo Chemical, Japan). Cells were plated on confocal dishes. After treatment, the live cells were stained with SiRhoNox-1 (5 *μ*M) for 1 h at 37°C. The medium was replaced with observation buffer, followed by observation with a confocal microscope (LSM710, Carl Zeiss, Germany). The excitation/emission used for SiRhoNox-1 was 635/660 nm.

### 2.7. Transferrin Endocytosis Assay

Cells were plated on confocal dishes. After treatment, the live cells were placed on ice for 10 min and then stained with pHrodo™ Red transferrin conjugate (P35376, Thermo Fisher Scientific, USA) for 20 min at 37°C. The medium was replaced with observation buffer, followed by observation with a confocal microscope (LSM710, Carl Zeiss, Germany). The excitation/emission used for pHrodo™ Red was 560/585 nm.

### 2.8. Western Blot Analysis

The total cellular protein lysates were separated by 12% SDS–PAGE and transferred to polyvinylidene fluoride membranes (Millipore, USA). The membranes were incubated with specific antibodies against CA9 (1 : 1000), transferrin receptor (TrfR) (1 : 1000), and ferritin heavy chain (FTH1) (1 : 1000) at 4°C. GAPDH (1 : 5000) was used as an internal control. Anti-CA9 antibody was obtained from Proteintech (Wuhan, China). The other antibodies were purchased from Cell Signaling Technology (Beverly, MA, USA).

### 2.9. Enzyme-Linked Immunosorbent Assay (ELISA)

Quantification of plasma CA9 in the clinical samples was performed by ELISA. Peripheral blood was collected and plasma was isolated by centrifugation at 4°C (2000 × g, 10 min). The isolated plasma was stored at −20°C until the assay. The plasma samples were diluted (1 : 20) with ELISA buffer and analyzed by using CA9 Human ELISA Kit (EHCA9, Thermo Fisher Scientific, USA) according to the manufacturer instructions. The absorbance was measured at 450 nm.

### 2.10. RNA Isolation and Quantitative Real-Time PCR Analysis

Total RNA was extracted from tissues or cells with TRIzol reagent (Invitrogen, USA). The isolated RNA (1.0 *μ*g) was reverse-transcribed into cDNA using random primers with a reverse transcription kit (R047A, Takara, Japan) according to the manufacturer's instructions. Real-time PCR analyses were performed with SYBR Green (R420A, Takara, Japan). For paired samples, the results were normalized to the expression of GAPDH (as an internal reference) and calculated according to the 2^−*ΔΔ*CT^ method [25]. For unpaired samples, the relative expression was compared by *Δ*CT. Specific primer sequences were as follows: human CA9, forward, 5′-CAGCACAGAAGGGGAACCAA-3′; reverse, 5′-GAGCAGGACAGGACAGTTACC-3′; human PTGS2, forward, 5′-CGGTGAAACTCTGGCTAGACAG-3′, reverse, 5′-GCAAACCGTAGATGCTCAGGGA-3′; human GAPDH, forward, 5′-AGCCACATCGCTCAGACAC-3′; reverse, 5′- GCCCAATACGACCAAATCC-3′.

### 2.11. Transfection of Cell Lines and In Vitro Lentivirus Infection

Full length of CA9 gene was inserted into the EX-Z5727-M02 vector (GenePharma, China) to construct the CA9 overexpression plasmid. The empty EX-NEG-M02 vector was used as the control (GenePharma, China). To knock down CA9, specific short hairpin RNA (shRNA) sequences were inserted into the pGPU6/GFP/Neo vector (GenePharma, China), and the sh-CA9#1 and sh-CA9#2 plasmids were generated. The shRNA target sequences were as follows: sh-CA9#1, 5′-GCCTATGAGCAGTTGCTGT-3′; sh-CA9#2, 5′-TCGCGTTCCTTGTGCAGAT-3′; sh-NC, 5′-TTCTCCGAACGTGTCACGT-3′. PGPU6/GFP/Neo-shNC (sh-NC) was used as the control (GenePharma, China). The plasmids were transfected with X-treme GENE HP DNA transfection reagent (Roche, Switzerland). Typically, cells were seeded into 6-well plates and transfected the next day with 2 *μ*g/well of specific plasmids. 48 h posttransfection, the cells were harvested and processed for the following experiments.

To label gefitinib-resistant cells, cells were transduced with lentiviral vectors encoding eGFP (LPP-NEG-Lv201-025-C, GeneCopoeia, China) at a multiplicity of infection of 20. Cells were subjected to puromycin (2 *μ*g/mL) selection after 48 h of transduction. EGFP fluorescence was detected by flow cytometry with the FITC channel.

### 2.12. Xenograft Mouse Model Assay

Five-week-old male BALB/c mice were maintained under specific pathogen-free conditions and manipulated according to protocols approved by the Institutional Animal Care and Use Committee (IACUC). PC9/GR cells and PC9 cells were mixed at a ratio of 9 : 1 and subcutaneously injected into mice in a 100 *μ*L volume. Tumor growth was examined every 3 days. When the tumor volumes (0.5 × length × width^2^) reached an average of 100 mm^3^, mice were treated as follows: (a) saline by oral gavage; (b) gefitinib (25 mg/kg) daily by oral gavage; (c) U104 (19 mg/kg) daily by intraperitoneal injection; (d) cisplatin (4 mg/kg) on days 1, 7, 14, and 21 by intraperitoneal injection; (e) U104 (19 mg/kg) daily combined with cisplatin (4 mg/kg) on days 1, 7, 14, and 21 by intraperitoneal injection. The tumors were resected after 28 days. Tumor weights were measured, and tumor tissues were stained with haematoxylin-eosin (HE) and immunohistochemistry (IHC).

### 2.13. Bioinformatics Analysis and Statistical Analysis

The publicly available gefitinib-resistant and parental cell line RNA sequencing (RNA-seq) datasets (GSE34228, GSE38310, GSE83666, and GSE112274) were obtained from the GEO database (https://www.ncbi.nlm.nih.gov/geo). Ferroptosis-related genes were obtained from the FerrDb database (http://www.zhounan.org/ferrdb) [26]. RNA-seq, mutation, methylation, copy number (GISTIC output), and clinical data from 574 patients with lung adenocarcinoma were derived from The Cancer Genome Atlas (TCGA) portal (https://portal.gdc.cancer.gov). RNA-seq and clinical data from 305 patients with lung adenocarcinoma were accessed from cBioPortal (http://www.cBioPortal.org/). Protein expression data of TCGA was accessed from Clinical Proteomic Tumor Analysis Consortium (CPTAC) portal (https://proteomics.cancer.gov/programs/cptac). All R-based analyses were conducted using R v4.1.0. R packages were used as follows: “Limma,” “UpSetR,” “VennDiagram,” “Survival,” and “Survminer”. Protein–protein interaction analysis was performed via the STRING tool (http://string-db.org/).

Statistical analysis was performed with GraphPad Prism 8.3.0 (GraphPad Software, USA). For parametric data, *t*-test (two-sided) or one-way ANOVA was performed. For comparison of multiple groups with repeated measures, two-way ANOVA with Bonferroni correction was performed. The mean values and the standard deviation (SD) are presented. Statistically significant differences are designated as follows: ^∗^*P* < 0.05, ^∗∗^*P* < 0.01, and ^∗∗∗^*P* < 0.001. All experiments were repeated at least three times.

## 3. Results

### 3.1. CA9 Is Upregulated in Gefitinib-Resistant Lung Cancer

**Figure 1:**
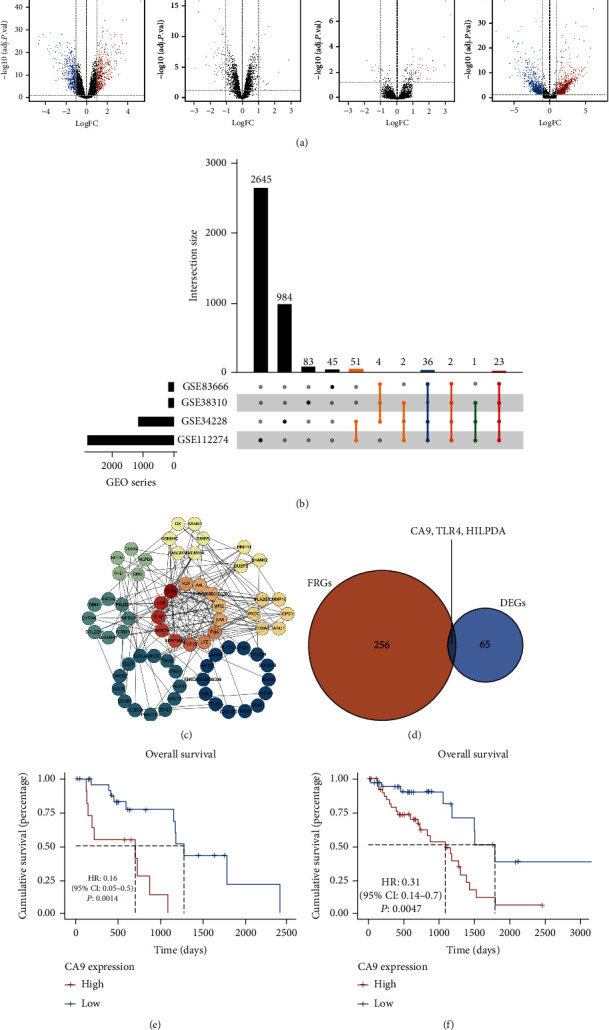
Systematic bioinformatics analysis identifies CA9 is upregulated in gefitinib-resistant lung cancer. (a) Differential expression analysis was performed in four RNA-seq datasets between gefitinib-sensitive and gefitinib-resistant cell lines (logFC > 1, *P* < 0.05) from the GEO database. (b) Coupregulated or codownregulated genes were screened in two or more datasets. (c) Protein–protein interaction analysis was performed via the STRING tool to summarize the protein interactions between the dysregulated protein-coding genes. (d) Wayne figure showing the intersection of the differentially expressed genes (DEGs) and ferroptosis-related genes (FRGs). (e, f) Survival analysis was performed in EGFR-mutant lung adenocarcinoma patients in the training set ((e), *n* = 39) and the testing set ((f), *n* = 76) from the TCGA database.

To explore the genetic alterations in gefitinib-resistant cancer cells, which may contribute to the vulnerability to ferroptosis, we performed a systematic bioinformatics analysis. As shown in Figure 1(a), differential expression analysis was performed in four RNA-seq datasets between gefitinib-sensitive and gefitinib-resistant cell lines (log2 fold change (logFC) > 1, *P* < 0.05). Coupregulated or codownregulated genes were screened in two or more datasets (Figure 1(b)). Protein–protein interaction networks were constructed to analyze protein interactions between the dysregulated protein-coding genes, and 68 differentially expressed genes (DEGs) were identified (Figure 1(c) and Supplementary Table S1). Finally, three genes were obtained by taking the intersection of the DEGs and the ferroptosis-related genes (FRGs) from the FerrDb database [26] (Figure 1(d) and Supplementary Table S2).

Then, we performed survival analysis to explore the effect of these dysregulated genes on prognosis. Only the ferroptosis suppressor gene CA9 was identified to be upregulated in gefitinib-resistant cells and predicted poor prognosis in 39 EGFR-mutant lung adenocarcinoma patients (the training set) from the TCGA database (Figure 1(e) and Supplementary Figure S1 (a, b)). We further confirmed that the upregulated gene CA9 was related to poor prognosis in 76 EGFR-mutant lung adenocarcinoma patients (the testing set) from the TCGA database (Figure 1(f)). We analyzed the relationship between CA9 mRNA expression level and tumor stage or histological grade in 305 lung adenocarcinoma patient samples [27]. The results showed that CA9 expression was not associated with tumor stage or histological grade (Supplementary Figures S1 (c, d)), which indicates that high CA9 expression is a poor prognostic factor in lung cancer patients independent of tumor stage or grade. We also performed multiomic analysis using the TCGA data to figure out the mechanism underlying CA9 upregulation. As shown in Supplementary Figure S1(e) and Supplementary Table S3, no correlations were observed between CA9 expression and mutations. Despite the positive relationship between CA9 expression and copy number, the correlation is weak (Supplementary Figure S1(e), correlation coefficient (*r*) = 0.115, *P* < 0.01). Then, we compared the correlations between CA9 expression and DNA methylation status. CA9 expression showed a strong negative correlation with methylation level of the CpG site cg20610181 (Supplementary Figure S1(e), *r* = −0.658, *P* < 0.001). Therefore, we inferred that DNA demethylation could be the underlying mechanism of CA9 upregulation.

**Figure 2:**
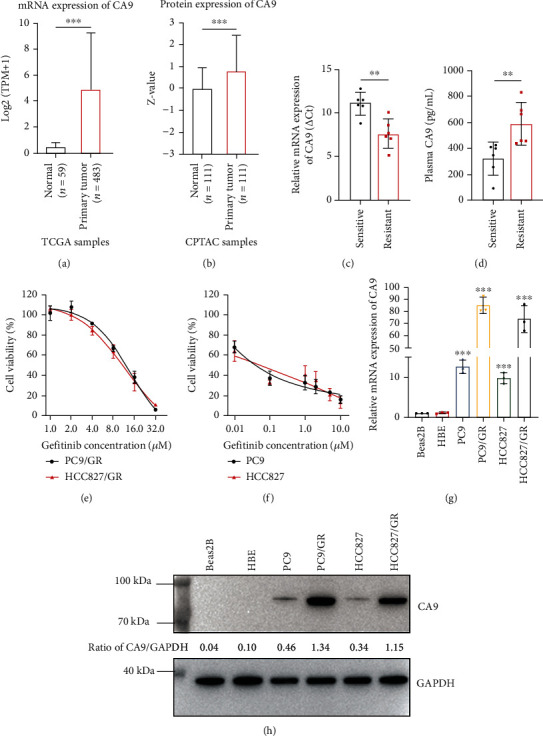
CA9 is upregulated in gefitinib-resistant cell lines and clinical samples. CA9 mRNA and protein expression levels were compared between nontumor tissues and lung cancer tissues in the TCGA database (a) and the CPTAC database (b). qPCR and ELISA were performed to investigate CA9 expression in lung cancer tissues (c) and plasma (d) from patients who benefitted from gefitinib (*n* = 6) and patients who acquired gefitinib resistance (*n* = 6). Smaller *Δ*CT values indicate higher mRNA levels. (e, f) Gefitinib sensitivity in parental (PC9 and HCC827) and gefitinib-resistant (PC9/GR and HCC827/GR) cells were analyzed by CCK8 assay. Expression of CA9 mRNA (g) and protein (h) in normal human bronchial epithelial cells (Beas2B and HBE), parental, and gefitinib-resistant cells was assessed by qPCR and western blot. Data shown are the Mean ± SDs of three independent experiments unless specified. (^∗∗^*P* < 0.01, ^∗∗∗^*P* < 0.001, Student's *t*-test).

Compared to the nontumor tissues, we demonstrated significantly higher CA9 mRNA expression using the TCGA database (*P* < 0.001; Figure 2(a)) and higher CA9 protein expression using the CPTAC database (*P* < 0.001; Figure 2(b)) in lung cancer tissues. Then, we measured CA9 expression in lung cancer tissues biopsied from patients who benefitted from gefitinib and patients who acquired gefitinib resistance. The clinical characteristics of patients are summarized in Supplementary Table S4. The results suggest that CA9 mRNA levels were commonly upregulated in gefitinib-resistant lung cancer tissues compared with sensitive tissues (Figure 2(c)). Also, the CA9 protein levels were higher in the plasma collected from patients who acquired gefitinib resistance (Figure 2(d)). We established gefitinib-resistant cells (PC9/GR and HCC827/GR) by exposing EGFR-mutant PC9 and HCC827 human lung cancer cells to increasing concentrations of gefitinib. In the CCK8 assay, PC9/GR and HCC827/GR cells survived in the presence of high-dose gefitinib (Figure 2(e)), whereas parental PC9 and HCC827 cells were sensitive to gefitinib (Figure 2(f)). Then, CA9 expression was evaluated in the gefitinib-resistant and parental cell lines as well as normal human bronchial epithelial cell lines (Beas2B and HBE). The expression levels of CA9 were higher in parental cell lines than in normal human bronchial epithelial cell lines. Furthermore, the gefitinib-resistant cell lines showed much higher CA9 mRNA and protein expression (Figures 2(g) and 2(h)). We challenged these cells with gefitinib and measured mRNA expression levels of CA9. As we speculated, the expression of CA9 in resistant cells gradually increased in time- and dose-dependent manner (Supplementary Figures S2 (a, b)). However, the CA9 expression in normal human bronchial epithelial cell lines (Supplementary Figures S2 (c, d)) and parental cell lines remained unaffected by gefitinib (Supplementary Figures S2 (e, f)).

### 3.2. CA9 Confers Resistance to Ferroptosis in Gefitinib-Resistant Cells

**Figure 3:**
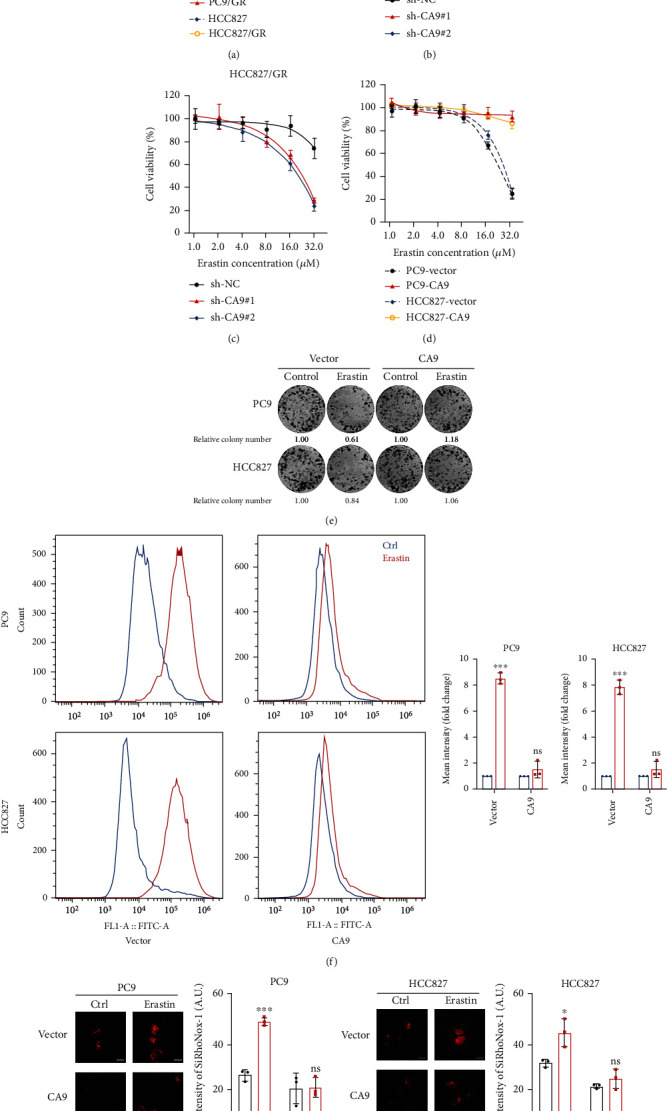
CA9 confers resistance to ferroptosis in gefitinib-resistant cells. (a) Erastin sensitivity in parental (PC9 and HCC827) and gefitinib-resistant (PC9/GR and HCC827/GR) cells were analyzed by CCK8 assay to reflect ferroptosis sensitivity. CA9-knockdown resistant cells (b, c) and CA9-overexpressing parental cells (d) were treated with erastin; cell viability was measured by CCK8 assay. (e) Long-term colony formation of CA9-overexpressing parental cells in the treatment of erastin (10 *μ*M). (f) Intracellular ROS were measured by flow cytometry (FITC channel) in CA9-overexpressing parental cells after erastin (10 *μ*M) treatment for 48 h. (g, h) Intracellular labile iron were measured by immunofluorescence staining in CA9-overexpressing parental cells after erastin (10 *μ*M) treatment for 48 h. The Mean ± SDs of three independent experiments are shown. (*ns* indicates not significant, ^∗^*P* < 0.05, ^∗∗∗^*P* < 0.001, Student's *t*-test).

A recent study reported that CA9 was involved in malignant mesothelioma resistance to ferroptosis under hypoxia [17]. Whether CA9 regulates ferroptosis sensitivity in gefitinib-resistant cells remains unknown. We challenged the parental and gefitinib-resistant cells with the widely used ferroptosis inducer, erastin. The parental cells were sensitive to erastin, in contrast, gefitinib-resistant cells were resistant to ferroptosis induction (Figure 3(a)). Knockdown of CA9 by shRNAs sensitized resistant cells to erastin (Figures 3(b) and 3(c) and Supplementary Figures S3 (a, b)), while overexpression of CA9 decreased the sensitivity of erastin in the parental cells (Figure 3(d) and Supplementary Figure S3(c)). In addition, the long-term colony formation assay indicated that the proliferation inhibition effect of erastin in parental cells was reduced by CA9 overexpression (Figure 3(e)). Previous study reported that CA9 is involved in erastin-induced ferroptosis [17]. However, erastin treatment did not affect the mRNA expression of CA9 in the resistant cells PC9/GR and HCC827/GR (Supplementary Figures S3(d)).

It is well known that ferroptosis is a form of cell death characterized by intracellular redox state imbalance and ROS elevation [9]. Thus, we measured ROS levels after erastin treatment. In parental cells, erastin induced ROS accumulation. Whereas, the erastin induced ROS elevation was markedly attenuated by CA9 overexpression in parental cells (Figure 3(f)). Since ferroptosis is a form of cell death that is iron-dependent [9], an increased intracellular labile iron pool is a hallmark of ferroptosis. Thus, we measured intracellular labile iron by immunofluorescence staining. As shown in Figures 3(g) and 3(h), elevated labile iron was detected after erastin treatment, while this effect was abolished by overexpression of CA9.

We also examined whether CA9 was involved in gefitinib resistance. We challenged the parental cells PC9 with gefitinib as well as Fer-1 (a specific ferroptosis inhibitor), DFO (an iron chelator), and z-VAD (an apoptosis inhibitor). Z-VAD rescued cell death induced by gefitinib in parental cells, but Fer-1 and DFO failed (Supplementary Figure S3(e)). Moreover, overexpression of CA9 did not affect gefitinib sensitivity in parental cells (Supplementary Figure S3 (f, g)). Collectively, these data suggest that CA9 confers resistance to erastin-induced ferroptosis rather than resistance to gefitinib.

### 3.3. CA9 Inhibition Triggers Ferroptosis in Gefitinib-Resistant Cells

**Figure 4:**
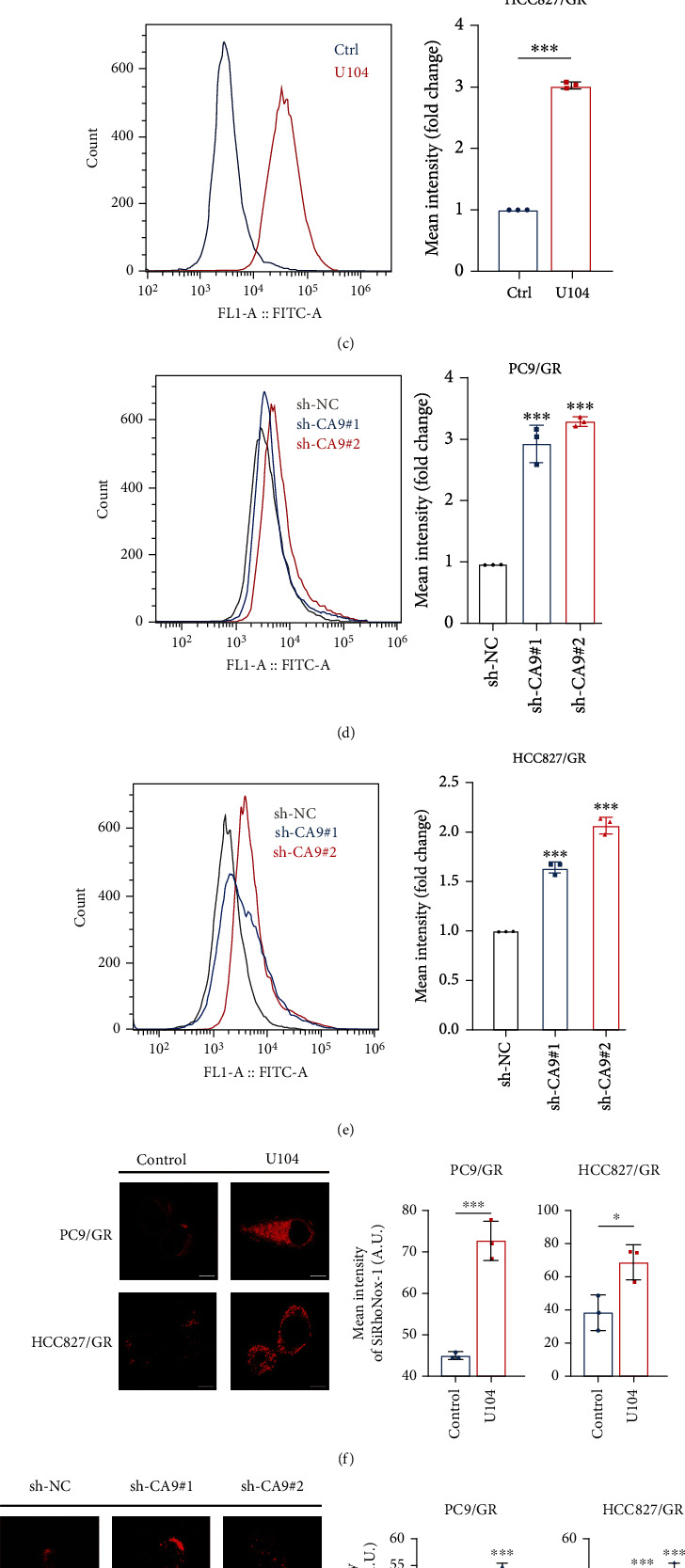
CA9 inhibition triggers ferroptosis in gefitinib-resistant cells. (a) Gefitinib-resistant PC9/GR cells were treated with CA9 inhibitor U104 (80 *μ*M) alone or combined with apoptosis inhibitor z-VAD (50 *μ*M), ferroptosis inhibitor Fer-1 (3 *μ*M), or iron chelator DFO (0.5 *μ*M) for 48 h. CCK8 assay was performed to measure cell viability. Intracellular ROS in gefitinib-resistant PC9/GR (b) and HCC827/GR (c) cells were measured by flow cytometry (FITC channel) after U104 (80 *μ*M) treatment for 48 h. Intracellular ROS in gefitinib-resistant PC9/GR (d) and HCC827/GR (e) cells were measured by flow cytometry (FITC channel) after CA9 knockdown. Intracellular labile iron in gefitinib-resistant cells was measured by immunofluorescence staining after U104 treatment for 48 h (f) or CA9 knockdown (g). The Mean ± SDs of three independent experiments are shown. (*ns* indicates not significant, ^∗^*P* < 0.05, ^∗∗^*P* < 0.01, ^∗∗∗^*P* < 0.001, Student's *t*-test).

To further investigate CA9's effect on ferroptosis regulation, we challenged the gefitinib-resistant cells PC9/GR with CA9 inhibitor, U104. U104 treatment triggered substantial cell death in PC9/GR cells. To confirm the ferroptotic cell death triggered by CA9 inhibition, Fer-1, DFO and z-VAD were cotreated with U104 in PC9/GR cells. Fer-1 and DFO rescued cell death induced by U104, while z-VAD only partially protected against U104-induced cell death (Figure 4(a)).

Then, we evaluated whether CA9 inhibition either by genetic or pharmacological approaches could affect the ROS levels in gefitinib-resistant cells. The results showed that the ROS levels were elevated after CA9 inhibition (Figures 4(b)–4(e)). Then, the levels of intracellular labile iron were detected after CA9 inhibition by immunofluorescence staining. Inhibiting CA9, whether by U104 treatment or CA9 knockdown, increased the labile iron levels in resistant cells (Figures 4(f) and 4(g)). Overall, inhibiting CA9 could trigger ferroptosis in gefitinib-resistant cells.

### 3.4. CA9 Controls Vulnerability to Ferroptosis through Regulation of Iron Metabolism

**Figure 5:**
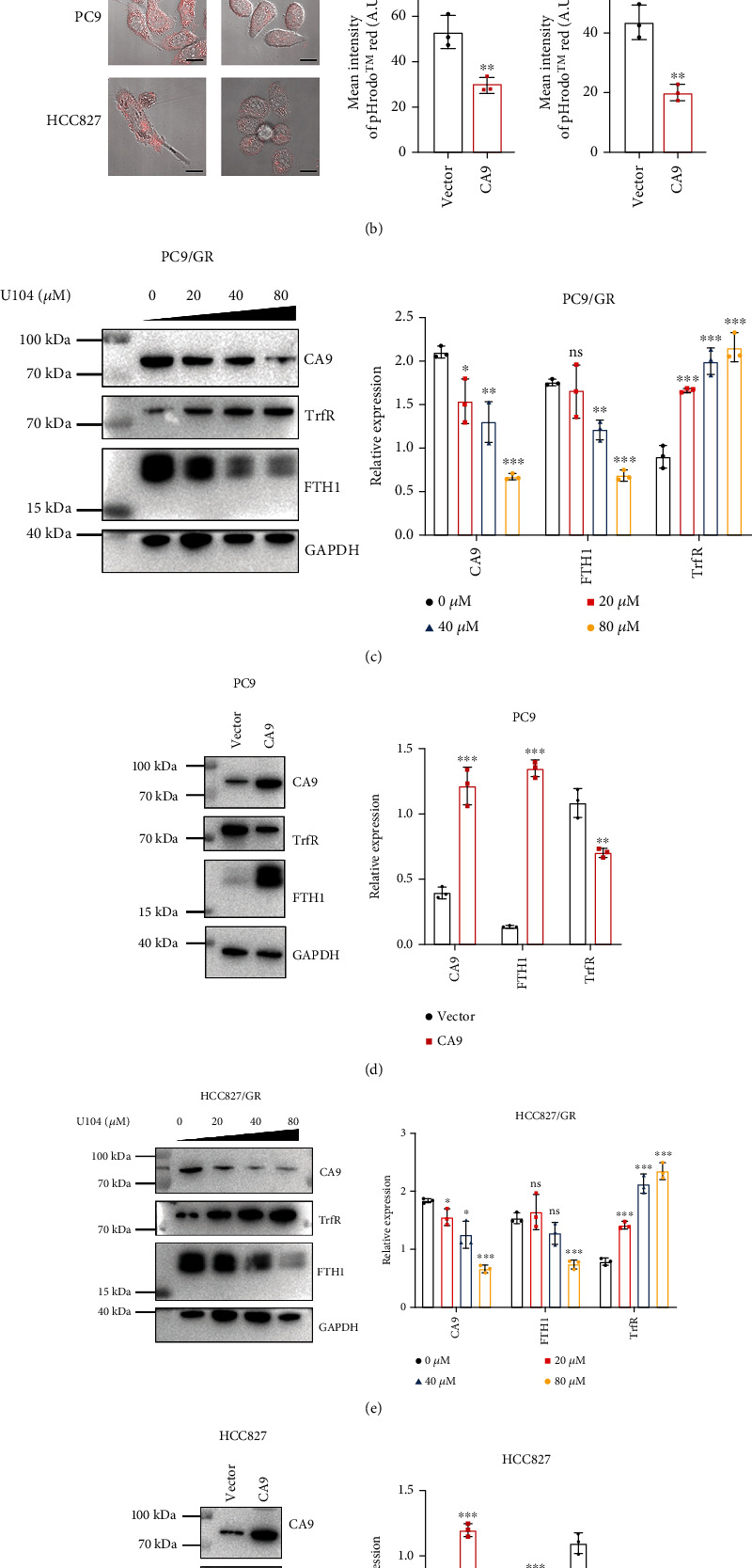
CA9 controls vulnerability to ferroptosis through regulation of iron metabolism. Transferrin endocytosis was detected by live-cell microscopy experiments with pH-sensitive pHrodo™ Red in gefitinib-resistant cells (PC9/GR and HCC827/GR) after U104 (80 *μ*M) treatment for 48 h (a) and in parental cells (PC9 and HCC827) after CA9 overexpression (b). Once internalized within endocytic vesicles, the labelled transferrin will be fluorescent (pink) and detectable in acidic environments. Protein lysates were harvested from gefitinib-resistant cells after U104 (80 *μ*M) treatment for 48 h (c, e) and parental cells after CA9 overexpression (d, f). Western blot analysis was performed for transferrin receptor (TrfR) as an iron uptake marker and ferritin heavy chain (FTH1) as an iron storage marker. The Mean ± SDs of three independent experiments are shown. (*ns* indicates not significant, ^∗^*P* < 0.05, ^∗∗^*P* < 0.01, ^∗∗∗^*P* < 0.001, Student's *t*-test).

As an iron-dependent form of cell death, iron metabolism plays an essential role in ferroptosis regulation [28]. We found elevated intracellular labile iron after CA9 inhibition (Figures 4(f) and 4(g)), which indicates that iron metabolism might be involved in ferroptosis regulation by targeting CA9. The transmembrane protein CA9 has a similar property as heat shock proteins (HSPs) in cytoskeletal networks of tumor cells by regulating cytosolic filaments [29]. HSPs have been reported to affect the endocytosis of transferrin by regulating the cytoskeleton, thus affecting iron uptake and inhibiting ferroptosis [30]. CA9 may potentially affect the endocytosis of transferrin [31], thus affecting iron uptake by cancer cells [32]. Next, we tried to determine the effect of CA9 on transferrin endocytosis by live-cell microscopy experiments with pH-sensitive pHrodo™ Red. Once internalized within endocytic vesicles, the labelled transferrin will be fluorescent and detectable in acidic environments. As speculated, the endocytosis of transferrin was enhanced by CA9 inhibition (Figure 5(a) and Supplementary Figure 4(a)). Then, we overexpressed CA9 in parental cells and found that the endocytosis of transferrin was suppressed as expected (Figure 5(b)).

Ferritin is the major iron storage protein in all living organisms [33]. A previous study showed that the stability of ferritin is pH-dependent [34]. CA9 has been reported to exert important functions in stabilizing the intracellular pH of cancer cells [16]. Therefore, we hypothesized that CA9 may be involved in the stabilization of intracellular ferritin. To test this hypothesis, we detected ferritin levels in gefitinib-resistant cells after genetic or pharmacological inhibition of CA9. As CA9 was inhibited, the FTH1 (the subunit of ferritin) protein level gradually decreased (Figures 5(c) and 5(e) and Supplementary Figures S4 (b, c)). Meanwhile, the TrfR protein level was increased after CA9 inhibition, which confirmed the ability of CA9 to suppress iron uptake (Figures 5(c) and 5(e) and Supplementary Figures S4 (b, c)). In keeping with our hypothesis, the protein levels of TrfR were downregulated but the levels of FTH1 were upregulated after CA9 overexpression in parental cells (Figures 5(d) and 5(f)).

These results suggest that CA9 is involved in the inhibition of transferrin endocytosis and the stabilization of ferritin. Targeting CA9 promotes iron uptake and release, thus triggering gefitinib-resistant cell ferroptosis.

### 3.5. Targeting CA9 Enhances the Ferroptosis-Inducing Effect of Cisplatin on Gefitinib-Resistant Cells

Previous studies have reported that NSCLC patients who respond well to treatment with initial EGFR-TKI and later experience therapy failure, demonstrate a second response to EGFR-TKI retreatment after drug withdrawal, known as the drug holiday effect [35, 36]. This effect can be explained, at least in part, by the elimination of drug-resistant cells in the heterogeneous tumor cell populations [37]. Compared to the drug holiday, cisplatin-based chemotherapy appeared to be more efficient in eliminating resistant cells [15]. As a widely used ferroptosis inducer, cisplatin may play a role in eliminating drug-resistant cells by inducing ferroptosis [14]. Yet, the efficacy of cisplatin-based chemotherapy between initial EGFR-TKI and TKI retreatment remains poor. We suppose that targeting CA9 could enhance the ferroptosis-inducing effect of cisplatin in gefitinib-resistant cells.

**Figure 6:**
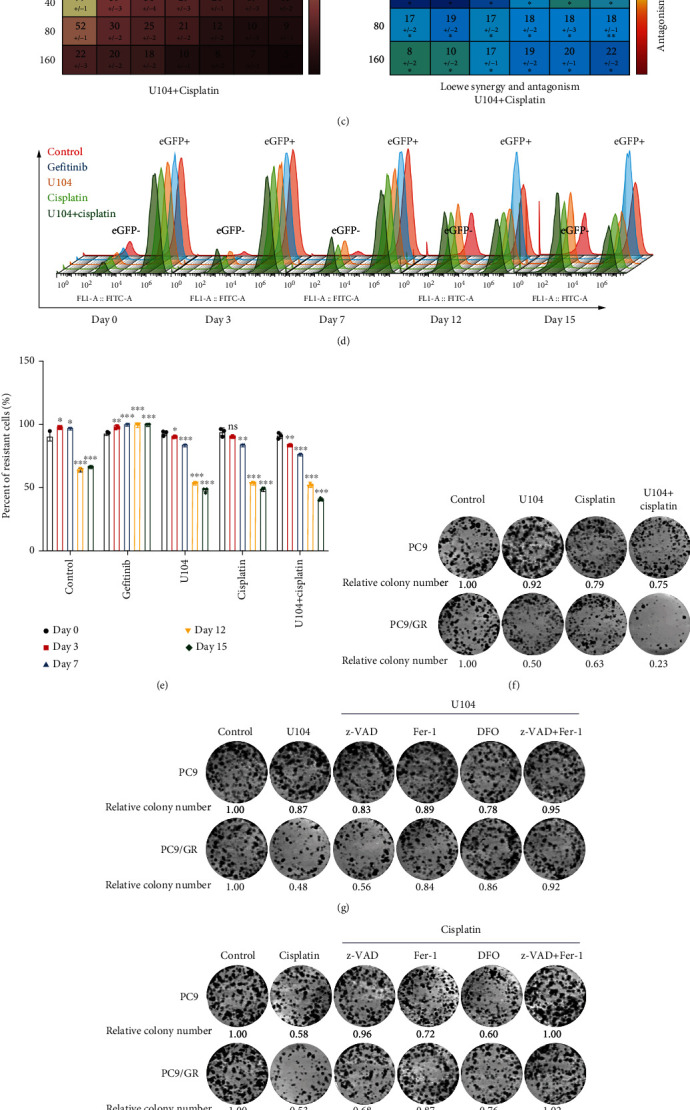
Targeting CA9 enhances the anticancer effect of cisplatin on gefitinib-resistant cells. (a, b) Long-term colony formation of CA9-knockdown parental and gefitinib-resistant cells. (c) Gefitinib-resistant PC9/GR cells were treated with different concentrations of CA9 inhibitor U104 and cisplatin for 48 h. A CCK8 assay was performed to measure cell viability. Shown is the heatmap of cell viability (%) at different combinations (left panel). A synergistic effect was measured by the Combenefit tool. Shown is the heatmap of synergy scores based on the Loewe excess additivity model (right panel). (d, e) Gefitinib-resistant PC9/GR cells were labelled with green by transduction with lentiviral vectors encoding eGFP. Then, gefitinib-resistant PC9/GR and parental PC9 cells were mixed at a ratio of 9 : 1 and treated with no drug, gefitinib (1 *μ*M), U104 (10 *μ*M), cisplatin (0.5 *μ*M), or U104 (10 *μ*M) + cisplatin (0.5 *μ*M) for 15 days. The relative abundance of the two populations was measured by flow cytometry (FITC channel). (f) PC9 parental and PC9/GR resistant cells were treated with U104 (10 *μ*M), cisplatin (0.5 *μ*M), or U104 (10 *μ*M) + cisplatin (0.5 *μ*M) in a long-term colony formation assay. (g, h) PC9 parental and PC9/GR resistant cells were treated with U104 (10 *μ*M), cisplatin (0.5 *μ*M) alone, or in combination with z-VAD (50 *μ*M), Fer-1 (3 *μ*M), DFO (0.5 *μ*M), or z-VAD (50 *μ*M) + Fer-1 (3 *μ*M) in the long-term colony formation assay. (i) Schematic of the in vitro competition assay to study the effect of CA9 inhibition in a heterogeneous tumor containing both gefitinib-resistant and gefitinib-sensitive cells. The Mean ± SDs of three independent experiments are shown. (*ns* indicates not significant, ^∗^*P* < 0.05, ^∗∗^*P* < 0.01, ^∗∗∗^*P* < 0.001, Student's *t*-test).

We first performed genetic silencing of CA9 using shRNAs in parental and gefitinib-resistant cells. In long-term proliferation assays, these shRNAs suppressed proliferation in gefitinib-resistant cells, but not in parental cells (Figures 6(a) and 6(b)). Our present data indicate that targeting CA9 is more efficient in gefitinib-resistant than in sensitive cells.

Then, we determined the synergistic effects of the CA9 inhibitor U104 and cisplatin in gefitinib-resistant cells. Drug-resistant cells were treated with different concentrations of U104 or cisplatin, and the synergistic scores were measured by the Combenefit software using data from the cell viability assays [38]. The results showed that U104 and cisplatin had significant synergistic effects in gefitinib-resistant cells (Figure 6(c)). The combination of 10 *μ*M U104 and 0.5 *μ*M cisplatin was selected for the next in vitro competition assay. Gefitinib-resistant cells were labelled with green by transduction with eGFP-encoding lentiviral vectors. Then, gefitinib-resistant and gefitinib-sensitive cells were mixed in a 9 : 1 ratio of resistant cells to sensitive cells. The mixed cells were treated with no drug, gefitinib, U104, cisplatin, or U104 + cisplatin. Over the following 15 days, the relative proportion of the two populations was tracked by flow cytometry (Figures 6(d) and 6(e)). The results showed that gefitinib enriched eGFP-positive gefitinib-resistant cells, while U104 or cisplatin efficiently depleted gefitinib-resistant cells. The tendency of the relative proportion changes in the U104 + cisplatin combination group was the same as in U104 or cisplatin single group. However, these changes were significant and started earlier.

Furthermore, we performed colony formation assay on parental and resistant cells under treatment of U104 or cisplatin. As shown in Figure 6(f), treatment of U104 inhibited the proliferation of resistant cells without affecting the parental cells. Cisplatin treatment showed stronger suppressive effects in resistant cells. The U104 + cisplatin combination showed significant synergistic effects in resistant cells rather than in parental cells. To determine the mode of cell death, we cotreated the parental and resistant cells with z-VAD, Fer-1, DFO as well as U104 and cisplatin. As expected, Fer-1 and DFO significantly rescued U104-mediated proliferation inhibition in resistant cells, while z-VAD could not (Figure 6(g)). Under cisplatin treatment, z-VAD showed stronger protective effects in parental cells but could only partially rescue the proliferation defect in resistant cells. Conversely, Fer-1 and DFO showed stronger protective effects in resistant cells (Figure 6(h)).

Collectively, these results indicate that targeting CA9 cooperates with cisplatin to eliminate gefitinib-resistant cells in the heterogeneous tumor cell populations by inducing ferroptosis, as shown in Figure 6(i).

### 3.6. Targeting CA9 Improves the Therapeutic Efficacy of Cisplatin in Gefitinib-Resistant Lung Cancer

**Figure 7:**
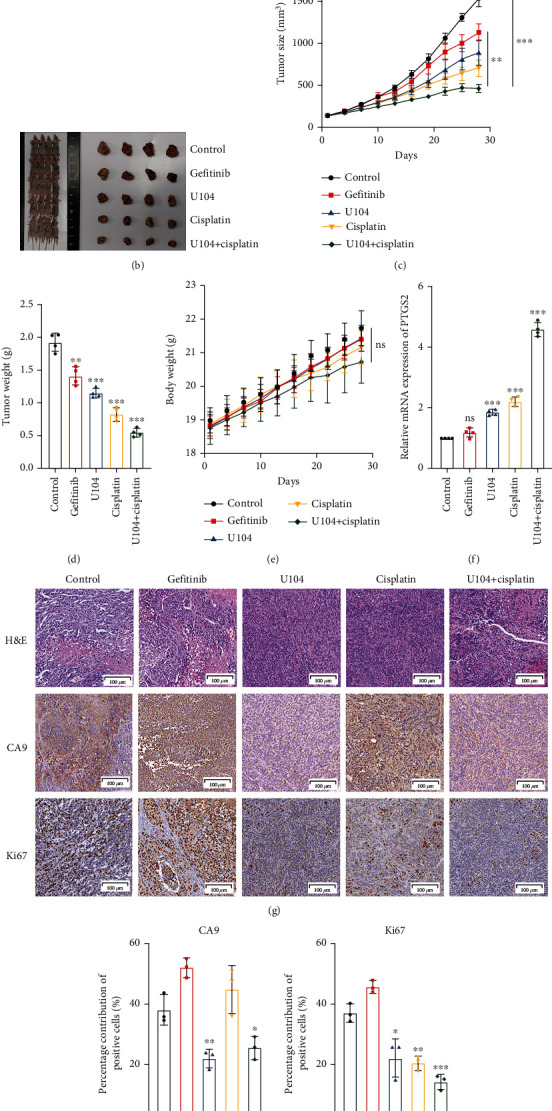
Targeting CA9 improves the therapeutic efficacy of cisplatin in gefitinib-resistant lung cancer. (a) A mixture of gefitinib-resistant PC9/GR and parental PC9 cells (PC9/GR: PC9, 9 : 1) were subcutaneously injected into BALB/c nude mice to model therapeutic targeting of heterogeneous tumor cell populations in vivo. (b) Mice were treated as follows: saline daily, gefitinib (25 mg/kg) daily, U104 (19 mg/kg) daily, cisplatin (4 mg/kg) on days 1, 7, 14, and 21, and U104 (19 mg/kg) daily combined with cisplatin (4 mg/kg) on days 1, 7, 14, and 21. Endpoint tumor images of the mice are shown. (c) Growth curves of tumors after different treatments. (^∗∗^*P* < 0.01, ^∗∗∗^*P* < 0.001, two-way ANOVA-RM with Bonferroni post hoc correction). (d) Tumor weights were compared at the endpoint. (^∗∗^*P* < 0.01, ^∗∗∗^*P* < 0.001, Student's *t*-test). (e) Body weights of mice were measured during treatments. (*ns* indicates not significant, two-way ANOVA-RM with Bonferroni correction). (f) qPCR analysis was performed for PTGS2 as a marker of ferroptosis in vivo. (g) Haematoxylin-eosin (HE) and immunohistochemical (IHC) staining were performed to evaluate the proliferative activity of tumors. (h) Quantification of CA9-positive cells. (i) Quantification of Ki67-positive cells. The data shown are the Mean ± SDs of four independent experiments. (^∗^*P* < 0.05, ^∗∗^*P* < 0.01, ^∗∗∗^*P* < 0.001, Student's *t*-test).

To model the therapeutic targeting of heterogeneous tumor cell populations in vivo, we mixed gefitinib-resistant PC9/GR cells together with gefitinib-sensitive PC9 cells and injected the admixture (PC9/GR: PC9, 9 : 1) subcutaneously in mice (Figure 7(a)) [39]. The mice were randomly divided into five groups and treated with saline, gefitinib, U104, cisplatin, or U104 + cisplatin to explore whether CA9 inhibition could improve the treatment effect of cisplatin. The results suggested that tumor growth was significantly inhibited in the U104 + cisplatin group (Figures 7(b)–7(d)) than that in the control group. No significant weight loss was observed in the combination group, indicating good tolerance to the combination therapy (Figure 7(e)). We further evaluated the mRNA expression of PTGS2, a marker for assessment of ferroptosis in vivo [40], finding that U104 combined with cisplatin significantly increased the PTGS2 expression in isolated tumor tissues (Figure 7(f)). CA9 staining indicated that CA9 expression was significantly suppressed in the combination group (Figures 7(g) and 7(h)). Ki67 staining demonstrated significantly reduced proliferative activity in the combination group (Figures 7(g) and 7(i)). Collectively, these findings indicate that the CA9 inhibitor, U104, reinforced the treatment effect of cisplatin by inducing ferroptosis on gefitinib-resistant xenograft tumors.

## 4. Discussion

Acquired resistance to EGFR-TKI in lung cancer remains a major problem to be solved in clinical practice. The subsequent treatment options after acquired resistance could be adopted depending on the resistance mechanisms [5–7]. However, for patients with acquired resistance mediated by an unknown mechanism, the therapeutic options remain limited. Recent studies have shown the promise of ferroptosis-inducing therapy in EGFR-TKI resistant cancer cells [10–12]. Even though several agents have been reported to induce ferroptosis, these ferroptosis inducers have not been translated into clinical benefits [13]. Here, we show that CA9 is upregulated in gefitinib-resistant lung cancer and confers resistance to ferroptosis-inducing drugs. Mechanistically, CA9 is involved in the inhibition of transferrin endocytosis and the stabilization of ferritin, leading to resistance to ferroptosis through regulation of iron metabolism. Targeting CA9 has been demonstrated to induce ferroptosis in gefitinib-resistant lung cancer.

Multiple studies have hinted that drug-resistant and sensitive clones coexist in heterogeneous tumor tissues. After EGFR-TKI treatment, the drug-sensitive cells are eliminated. Once the drug-resistant cells achieve dominant status in the heterogeneous tumor cell populations, clinical resistance develops. In the second-line of treatment, cisplatin-based chemotherapy targets these drug-resistant cells while sparing the sensitive cells. This means that subsequent rechallenge with EGFR-TKI could theoretically provide clinical benefit as the left cells still retain sensitivity to EGFR-TKI [41–43]. As the widely used ferroptosis inducer, cisplatin may play a role in eliminating drug-resistant cells by inducing ferroptosis [14]. A prospective, multicenter phase II study (RE-CHALLENGE, CTONG1304) has reported that patients who received first-line gefitinib treatment could still benefit from gefitinib retreatment after the second-line cisplatin-based chemotherapy. Median progression-free survival of these patients receiving third-line gefitinib treatment was 4.4 months and median overall survival was 10.3 months [15]. Despite the favorable efficacy data shown in this study, the long-term benefit remains poor. Exploring more effective therapeutic strategies to eliminate more gefitinib-resistant cells and to gain a longer drug holiday period is important.

Our present data indicate that CA9-targeting treatment is more efficient in gefitinib-resistant cells than in sensitive cells. Therefore, we explored the role of targeting CA9 in combination with cisplatin in inducing ferroptosis in gefitinib-resistant cells. Our study found that targeting CA9 has a significant synergistic effect with cisplatin in inhibiting the viability of gefitinib-resistant cells. The synergistic effect of targeting CA9 and cisplatin on the elimination of gefitinib-resistant cells was further verified through an in vitro competition assay. Moreover, the combined treatment with cisplatin and CA9 inhibitor U104 was much more effective than cisplatin alone in the xenograft model containing heterogeneous tumor cell populations. In our study, inhibiting CA9 triggers ferroptosis through regulation of iron metabolism. Cisplatin was reported to induce ferroptosis predominantly through the depletion of reduced glutathione and the inactivation of glutathione peroxidase [14]. Consistent with our study, CA9 regulation was not involved in cisplatin-induced ferroptosis. Of note, the U104 + cisplatin combination treatment promoted ferroptosis via two distinct pathways in tumor tissues. These findings indicate that CA9 is an actionable target for enhancing cisplatin efficacy in gefitinib-resistant lung cancer by inducing ferroptosis.

There are several limitations in this study. First, considering the heterogeneity of gefitinib resistance mechanisms, we performed our experiments in two types of gefitinib-resistant cell lines PC9/GR and HCC827/GR. However, it is still controversial whether the two resistant cell lines could faithfully recapitulate clinical drug resistance. Second, we injected the admixture of resistant and sensitive cells subcutaneously in mice to model the heterogeneous tumor cell populations. But, we were not able to evaluate the relative abundance of the two populations in vivo. At last, we only demonstrated that CA9-targeting therapy improved gefitinib-resistant lung cancer treatment. We also identified CA9 upregulation in osimertinib-resistant cell lines HCC827/OR (data not shown). Thus, further studies on whether CA9-targeting therapy improves osimertinib-resistant lung cancer treatment would be beneficial.

Altogether, this study found that CA9 is upregulated in gefitinib-resistant lung cancer and confers resistance to ferroptosis-inducing drugs. CA9-targeting therapy is a promising approach to improve the therapeutic effect of gefitinib-resistant lung cancer by inducing ferroptosis.

## Data Availability

All data and materials supporting the conclusions were included in the main paper.

## Supplementary Materials

Supplementary Materials Figure S1: TCGA analysis of dysregulated genes. Figure S2: qPCR analysis of CA9 expression after gefitinib treatment. Figure S3: CA9 expression does not affect gefitinib sensitivity in lung cancer cells. Figure S4: CA9 inhibition affects cellular iron metabolism. Table S1: 68 differential expressed genes (DEGs) identified by protein-protein interaction analysis. Table S2: 259 ferroptosis-related genes (FRGs) obtained from FerrDb database. Table S3: mutations in CA9 in the TCGA cohort. Table S4: the clinical characteristics of 12 NSCLC patients.
