# Supplementary material for: Carbonic Anhydrase IX Controls Vulnerability to Ferroptosis in Gefitinib-Resistant Lung Cancer
Source: Oxid Med Cell Longev. 2023 Jan 31;2023:1367938. doi: 10.1155/2023/1367938 (PMC9904911; doi:10.1155/2023/1367938)
Supplement: Supplementary Materials — Figure S1: TCGA analysis of dysregulated genes. Figure S2: qPCR analysis of CA9 expression after gefitinib treatment. Figure S3: CA9 expression does not affect gefitinib sensitivity in lung cancer cells. Figure S4: CA9 inhibition affects cellular iron metabolism. Table S1: 68 differential expressed genes (DEGs) identified by protein-protein interaction analysis. Table S2: 259 ferroptosis-related genes (FRGs) obtained from FerrDb database. Table S3: mutations in CA9 in the TCGA cohort. Table S4: the clinical characteristics of 12 NSCLC patients. [file 1367938.f1.docx]

**Supplementary Figures**


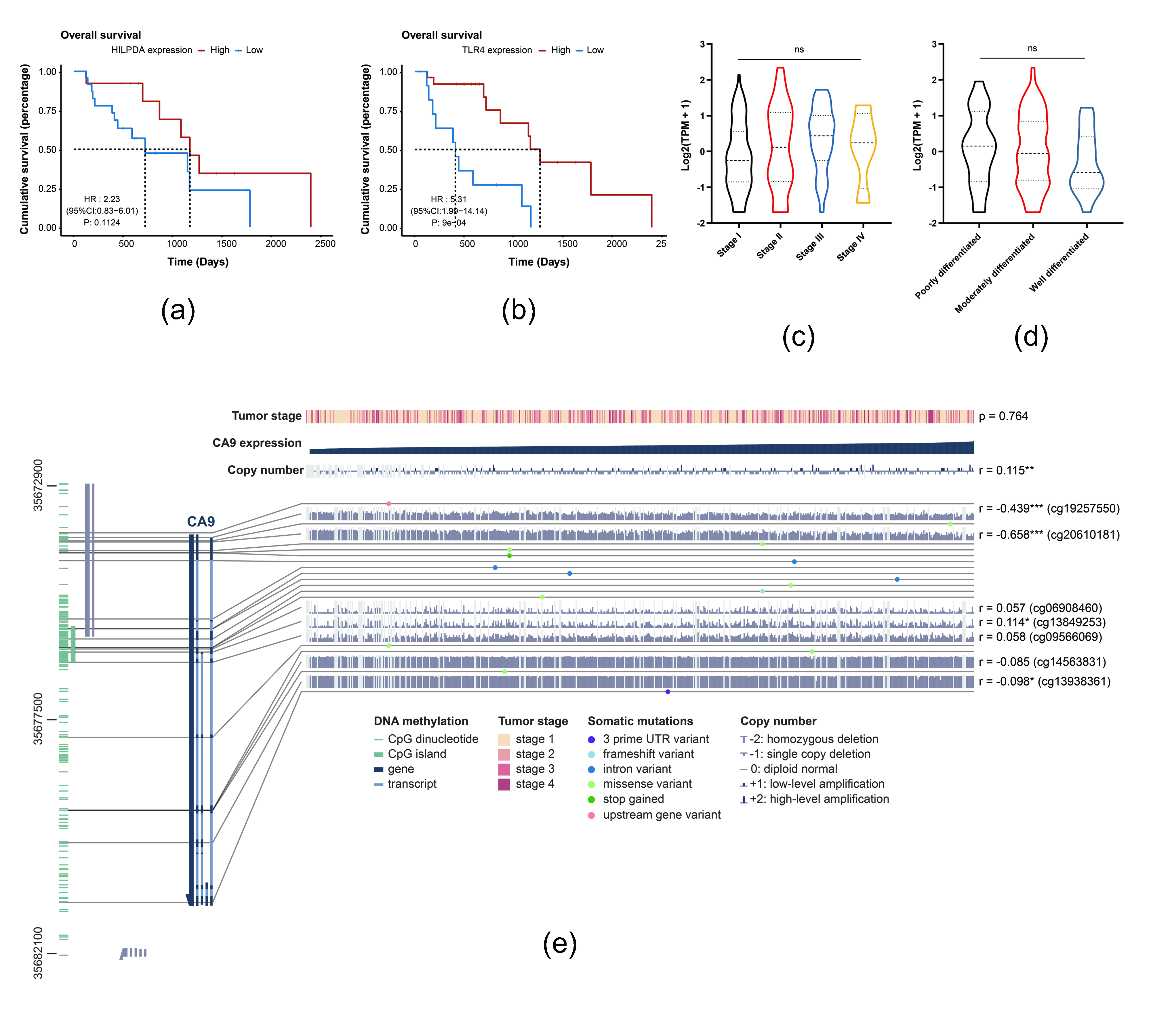
FIGURE S1: TCGA analysis of dysregulated genes. (a) Survival analysis indicated that the expression of HILPDA was not related to the progression-free survival (PFS) of EGFR-mutant lung adenocarcinoma patients from the TCGA database (*n*=39). (b) Low expression of TLR4 was associated with poor prognosis in EGFR-mutant lung adenocarcinoma patients (*n*=39), while TLR4 was upregulated in gefitinib-resistant cells. (c, d) CA9 expression was not correlated with lung cancer stage ((c)) or grade ((d)) (*ns* indicates not significant, one-way ANOVA with Bonferroni correction). (e) The correlations between CA9 expression and somatic mutations, copy number and DNA methylation status (**P*<0.05, ***P*<0.01, ****P*<0.001, Pearson’s correlation test).


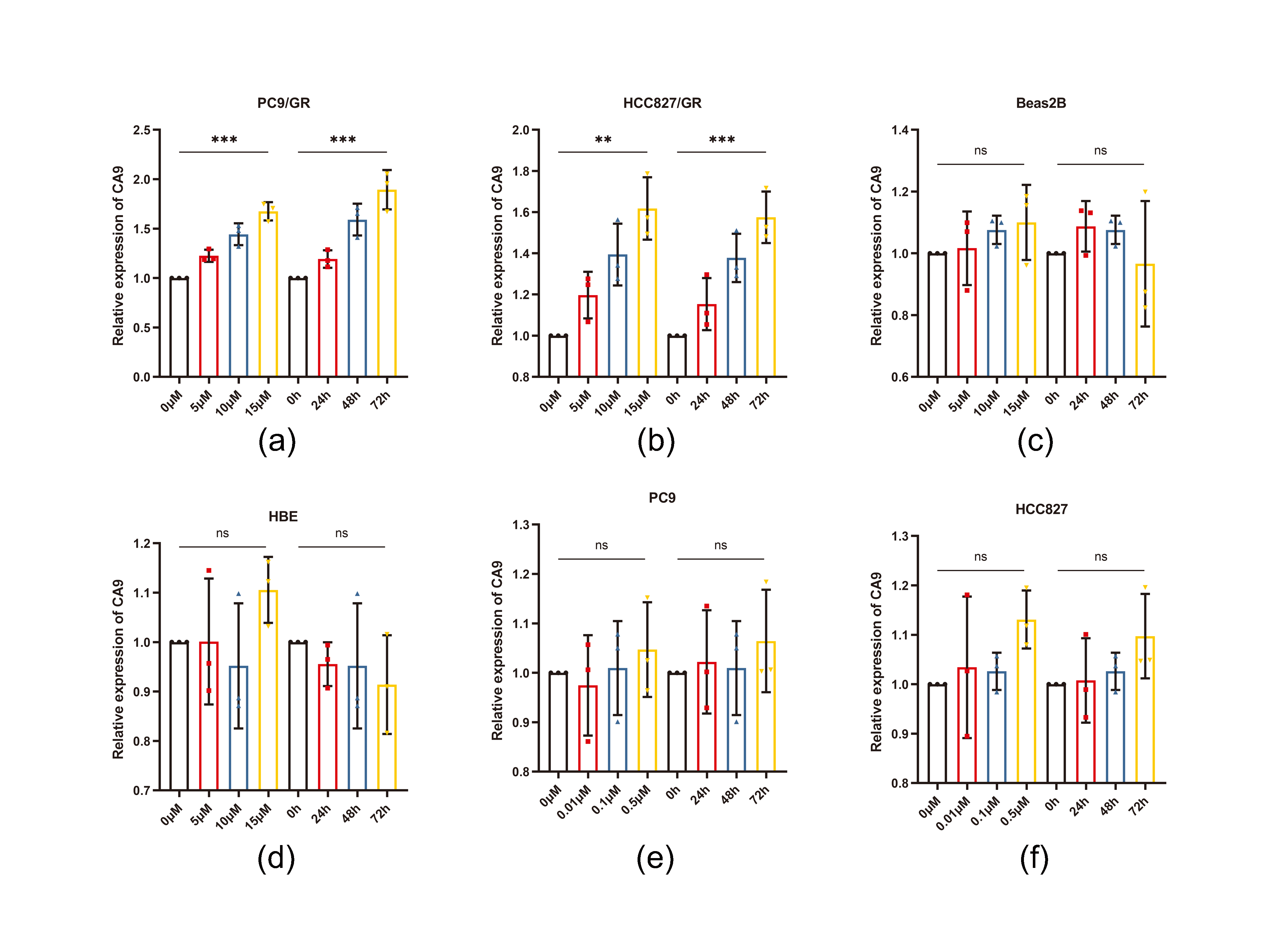
FIGURE S2: qPCR analysis of CA9 expression after gefitinib treatment. (a, b) CA9 expression in gefitinib-resistant cells PC9/GR((a)) and HCC827/GR((b)) gradually increased after gefitinib treatment in time- and dose-dependent manner. (c-f) CA9 expression in normal human bronchial epithelial cell lines ((c, d)) and parental cell lines ((e, f)) remained unaffected by gefitinib. The Mean±SDs of three independent experiments are shown. (*ns* indicates not significant, ***P*<0.01, ****P*<0.001, Student’s t-test).

**
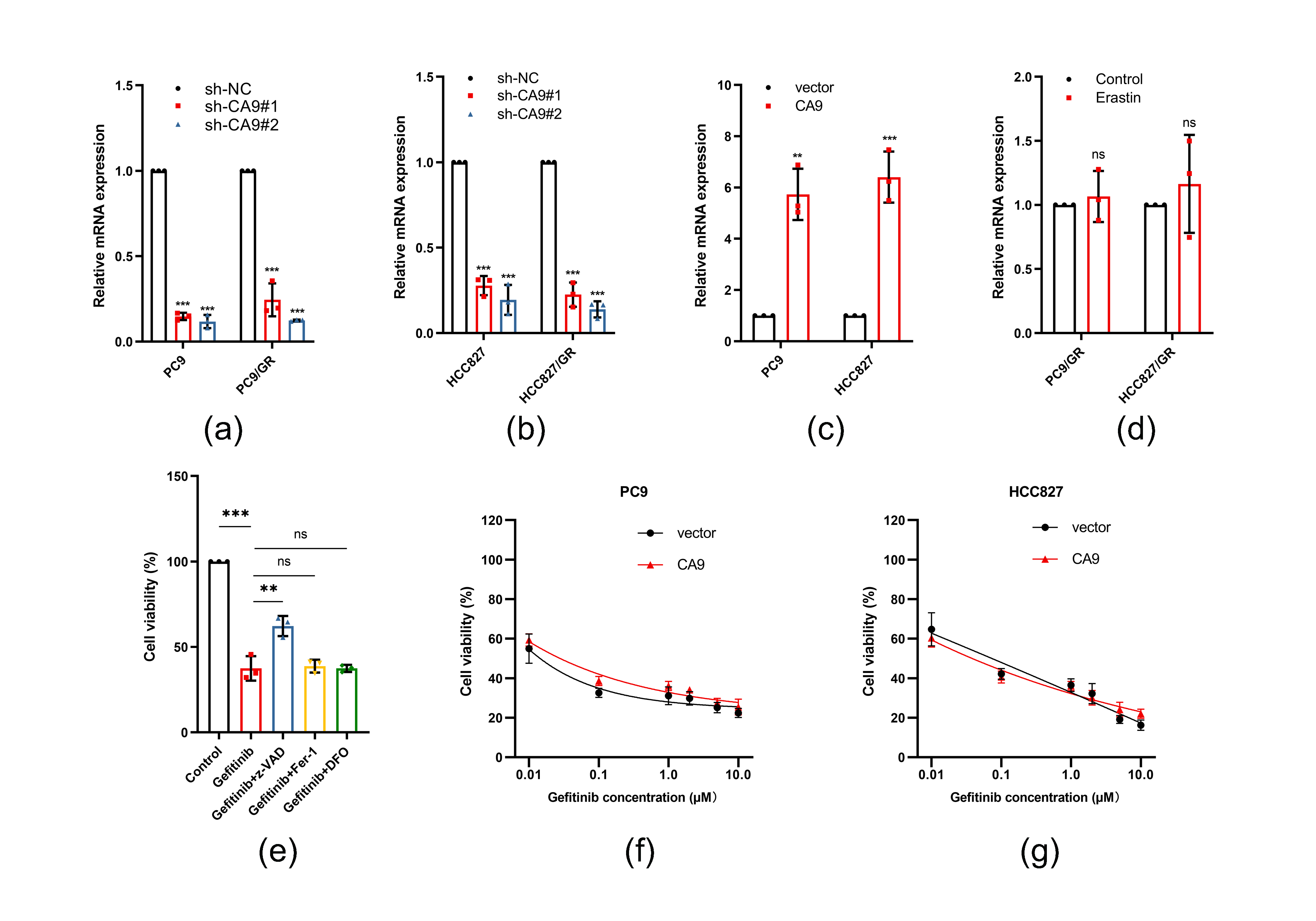
**

FIGURE S3: CA9 expression does not affect gefitinib sensitivity in lung cancer cells. (a, b) qPCR analysis of CA9 expression after CA9 knockdown in resistant cells. (c) qPCR analysis of CA9 expression after CA9 overexpression in parental cells. (d) qPCR analysis of CA9 expression after erastin (10 μM) treatment for 48 h. (e) PC9 parental cells were treated with gefitinib (1.0 μM) alone or combined with z-VAD (50 μM), Fer-1 (3 μM), or DFO (0.5 μM) for 48 h. CCK8 assay was performed to measure cell viability. (f, g) Gefitinib sensitivity in parental cells PC9 ((f)) and HCC827 ((g)) were analyzed after CA9 overexpression by CCK8 assay. The Mean±SDs of three independent experiments are shown. (*ns* indicates not significant, ***P*<0.01, ****P*<0.001, Student’s t-test).


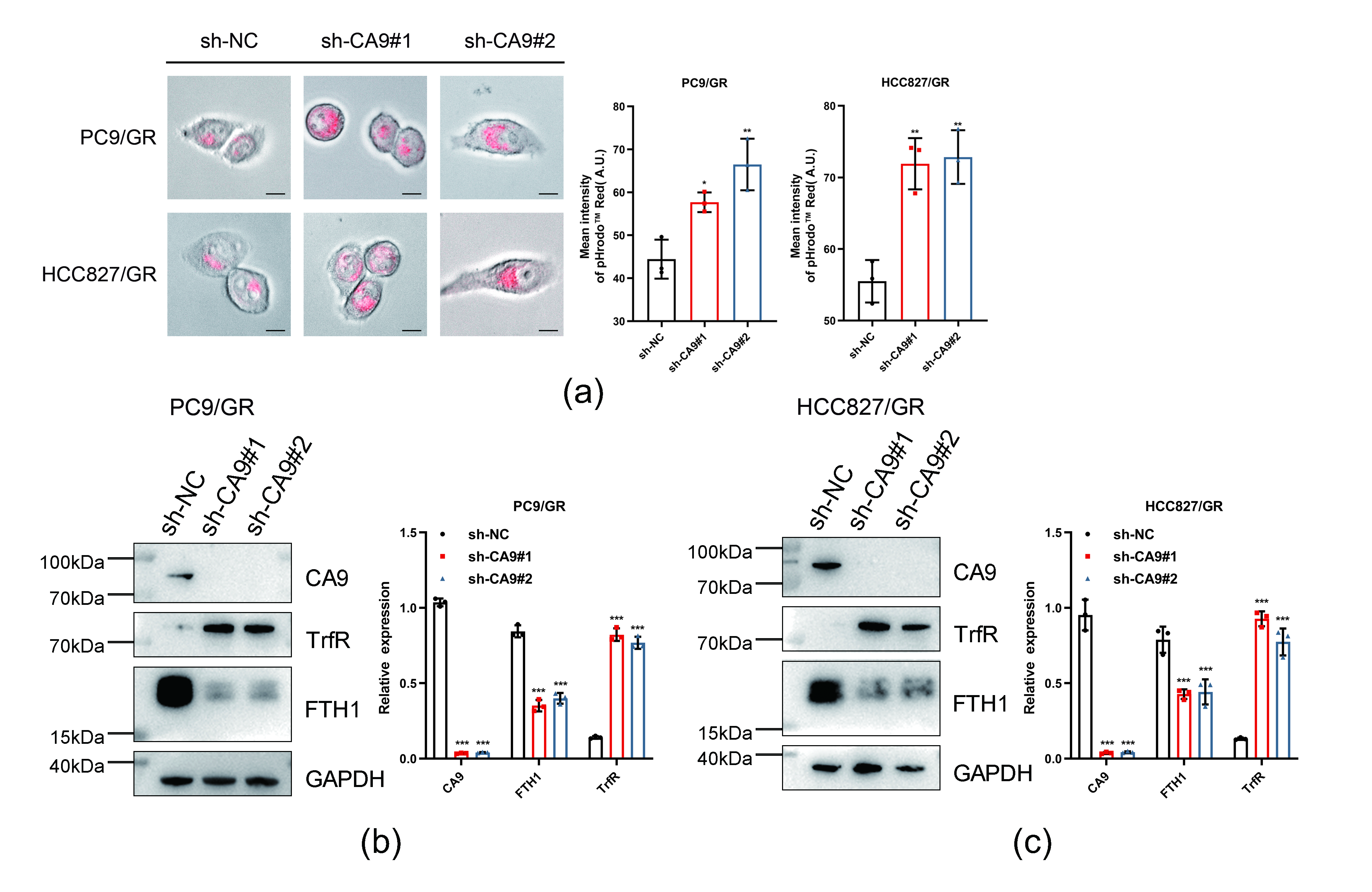


FIGURE S4: CA9 inhibition affects cellular iron metabolism. (a) Transferrin endocytosis was detected by live-cell microscopy experiments with pH-sensitive pHrodoTM Red in gefitinib-resistant cells (PC9/GR and HCC827/GR) after CA9 knockdown. Once internalized within endocytic vesicles, the labelled transferrin will be fluorescent (pink) and detectable in acidic environments. (b, c) Protein lysates were harvested from gefitinib-resistant cells PC9/GR ((b)) and HCC827/GR ((c)) after CA9 knockdown. Western blot analysis was performed for transferrin receptor (TrfR) as an iron uptake marker and ferritin heavy chain (FTH1) as an iron storage marker. The Mean±SDs of three independent experiments are shown. (*ns* indicates not significant, **P*<0.05, ***P*<0.01, ****P*<0.001, Student’s t-test).

**Supplementary Tables**

Table S1: 68 differential expressed genes (DEGs) identified by protein-protein interaction analysis.

| **Upregulated gene** | | | |
| --- | --- | --- | --- |
| TLR4 | DDX49 | NANOG | ACSS1 |
| LYZ | DBN1 | ENSG00000160200 | SGCB |
| MFI2 | PHACTR3 | FOXA2 | PRB1 |
| MMP16 | LOXL4 | MMP11 | TTC30A |
| RASGRP1 | AP4S1 | KANK4 | PID1 |
| NPTX1 | NRP2 | ADRB1 | FGF13 |
| CYTH4 | TLR2 | FBLN2 | CA9 |
| DDHD1 | VGF | CDHR3 | PLA2G7 |
| DLC1 | ARG1 | TNNI2 | TMEM45A |
| ZNF365 | DUSP5 | CACNG6 | FHL1 |
| ENSG00000269026 | GK | KLHDC1 | COL22A1 |
| SLC35F3 | WFDC6 | SERPINA1 | ADCY9 |
| LOX | KMT2C | AXL | LINGO2 |
| FGA | CC2D2A | PROC | ADAM2 |
| CPS1 | NKD2 | SIRPA | CGNL1 |
| SHANK2 | PAG1 | SMO | ZFYVE28 |
| CDKN1C | GABRP | BHLHE41 |  |
| **Downregulated gene** | | | |
| HILPDA | | | |

Table S2: 259 ferroptosis-related genes (FRGs) obtained from FerrDb database.

| **Ferroptosis-related gene** | | | |
| --- | --- | --- | --- |
| ABCC1 | DRD5 | LOC284561 | RIPK1 |
| ACO1 | DUOX1 | LOC390705 | RPL8 |
| ACSF2 | DUOX2 | LONP1 | RRM2 |
| ACSL3 | DUSP1 | LPCAT3 | SAT1 |
| ACSL4 | EGFR | LPIN1 | SCD |
| ACVR1B | EGLN2 | LURAP1L | SCP2 |
| AGPAT3 | EIF2AK4 | MAFG | SELENOS |
| AIFM2 | EIF2S1 | MAP1LC3A | SESN2 |
| AKR1C1 | ELAVL1 | MAP3K5 | SETD1B |
| AKR1C2 | EMC2 | MAPK1 | SIRT1 |
| AKR1C3 | ENPP2 | MAPK14 | SLC1A4 |
| ALB | EPAS1 | MAPK3 | SLC1A5 |
| ALOX12 | FADS2 | MAPK8 | SLC2A1 |
| ALOX12B | FANCD2 | MAPK9 | SLC2A12 |
| ALOX15 | FBXW7 | MIOX | SLC2A14 |
| ALOX15B | Fer1HCH | MIR137 | SLC2A3 |
| ALOX5 | FH | MIR17 | SLC2A6 |
| ALOXE3 | FLT3 | MIR212 | SLC2A8 |
| ANGPTL7 | FTH1 | MIR30B | SLC38A1 |
| ANO6 | FTL | MIR4715 | SLC3A2 |
| ARNTL | FTMT | MIR6852 | SLC40A1 |
| ARRDC3 | G6PD | MIR9-1 | SLC7A11 |
| ASNS | G6PDX | MIR9-2 | SLC7A5 |
| ATF3 | GABARAPL1 | MIR9-3 | SNORA16A |
| ATF4 | GABARAPL2 | MT1G | SNX4 |
| ATG13 | GABPB1 | MT3 | SOCS1 |
| ATG16L1 | GCH1 | MTDH | SP1 |
| ATG3 | GCLC | MTOR | SQSTM1 |
| ATG4D | GDF15 | MUC1 | SRC |
| ATG5 | GLS2 | MYB | SRXN1 |
| ATG7 | GLUT13 | NCF2 | STAT3 |
| ATM | GOT1 | NCOA4 | STEAP3 |
| ATP5MC3 | GPT2 | NF2 | STMN1 |
| ATP6V1G2 | GPX2 | NFE2L2 | TAZ |
| AURKA | GPX4 | NFS1 | TF |
| BACH1 | HAMP | NGB | TFAP2C |
| BAP1 | HBA1 | NNMT | TFR2 |
| BECN1 | HELLS | NOS2 | TFRC |
| BID | HERPUD1 | NOX1 | TGFBR1 |
| BLOC1S5-TXNDC5 | HIC1 | NOX3 | TLR4 |
| BNIP3 | HIF1A | NOX4 | TMBIM4 |
| BRD4 | HILPDA | NOX5 | TNFAIP3 |
| CA9 | HMGB1 | NQO1 | TP53 |
| CAPG | HMOX1 | NRAS | TP63 |
| CARS1 | HNF4A | OTUB1 | TRIB3 |
| CAV1 | HRAS | OXSR1 | TSC22D3 |
| CBS | HSD17B11 | PANX1 | TUBE1 |
| CD44 | HSF1 | PCK2 | TXNIP |
| CDKN1A | HSPA5 | PEBP1 | TXNRD1 |
| CDKN2A | HSPB1 | PGD | UBC |
| CDO1 | IDH1 | PHKG2 | ULK1 |
| CEBPG | IFNG | PIK3CA | ULK2 |
| CHAC1 | IL33 | PLIN2 | VDAC2 |
| CHMP5 | IL6 | PLIN4 | VEGFA |
| CHMP6 | IREB2 | PML | VLDLR |
| CISD1 | ISCU | PRDX1 | WIPI1 |
| CISD2 | JDP2 | PRDX6 | WIPI2 |
| CS | JUN | PRKAA1 | XBP1 |
| CXCL2 | KEAP1 | PRKAA2 | YWHAE |
| CYBB | KIM-1 | PROM2 | YY1AP1 |
| DDIT3 | KLHL24 | PSAT1 | ZEB1 |
| DDIT4 | KRAS | PTGS2 | ZFP36 |
| DNAJB6 | LAMP2 | RB1 | ZFP69B |
| DPP4 | LINC00336 | RELA | ZNF419 |
| DRD4 | LINC00472 | RGS4 |  |

Table S3: Mutations in CA9 in the TCGA cohort.

| **Mutation effect** | **Amino acid change** | **Sample** |
| --- | --- | --- |
| upstream gene variant | / | TCGA_L9_A7SV_01 |
| missense variant | p.P72H | TCGA_55_8506_01 |
| missense variant | p.E86D | TCGA_86_A4JF_01 |
| stop gained | p.E87* | TCGA_86_A4JF_01 |
| intron variant | / | TCGA_49_AAR4_01 |
| intron variant | / | TCGA_80_5607_01 |
| intron variant | / | TCGA_78_7158_01 |
| intron variant | / | TCGA_99_8033_01 |
| missense variant | p.L195R | TCGA_78_7150_01 |
| frameshift variant | p.P207Lfs*4 | TCGA_55_8506_01 |
| missense variant | p.P208A | TCGA_44_3918_01 |
| 3' prime UTR variant | / | TCGA_49_AARO_01 |

Table S4: The Clinical Characteristics of 12 NSCLC Patients.

| **Clinical characteristics** | **Sensitive group (N=6)** | **Resistant group (N=6)** |
| --- | --- | --- |
| Sex |  |  |
| Male | 2 | 2 |
| Female | 4 | 4 |
| Age, yr. |  |  |
| <65 | 3 | 3 |
| ≥65 | 3 | 3 |
| Histological type |  |  |
| Adenocarcinoma | 6 | 6 |
| Stage |  |  |
| IIIB | 4 | 3 |
| IV | 2 | 3 |
| EGFR mutation |  |  |
| Exon 19 deletion | 2 | 3 |
| Exon 21 L858R mutation | 4 | 3 |
| Resistance mechanism |  |  |
| MET amplification | / | 1 |
| ERBB2 amplification | / | 1 |
| No EGFR mutation was detected | / | 2 |
| unknown | / | 2 |
